# Urine non-contact sensor that evaluates multiple urination using capacitance change to inform when to replace inner diaper

**DOI:** 10.3389/fdgth.2025.1569144

**Published:** 2025-04-28

**Authors:** Keisuke Shichitani, Kazuki Nakajima

**Affiliations:** ^1^ Division of Bio-information Engineering, Department of Engineering, University of Toyama, Toyama, Japan; ^2^ Electronics and Device Technology Section, Toyama Industrial Technology Research and Development Center, Toyama, Japan

**Keywords:** diaper sensor system, capacitance, urination time, multiple urination, urine absorption volume

## Abstract

**Introduction:**

Using the toilet to excrete is important, which is a matter of human dignity. However, incontinent and bedridden elderly people who are unable to go to the toilet use disposable adult diapers. In this study, we developed a diaper sensor system that uses capacitance changes to quantitatively evaluate the volume of urine absorbed by diapers from different manufacturers and different approximate numbers of absorption (ANA) and to inform caregivers when to replace diapers.

**Methods:**

Three experiments, α: comparative verification by urination pattern, β: effect of ANA, and γ: evaluation by each diaper manufacturer, were conducted using a plastic torso filled with saline as a phantom. In addition, examine the actual urination time and urine absorption volume when the human wears a diaper; one volunteer wore inner and outer diapers, and pseudo-urine was infused via a tube.

**Results:**

Overall, the results indicate that the accuracy of urine absorption volume estimation showed that the pattern of multiple infusions followed that of a single infusion.

**Discussion:**

The results confirm that the diaper sensor system is highly versatile and independent of the urination pattern and diaper manufacturer. If the diaper sensor system is put into practical use, it is expected to provide quantitative information on when to replace diapers.

## 1 Introduction

Using the toilet to excrete is a crucial part of human pride and dignity (1). However, incontinent, bedridden, and physically disabled people who are unable to go to the toilet use disposable adult diapers, body-worn absorbent products, or absorbent incontinence aids (2). The urinary status of diaper users cannot be checked without removing their pants and opening the diaper. Bladder diaries are used in medical and nursing care to obtain urinary information, such as urination time and voided volume (3). They can be used to determine the timing of diaper changes, appropriate diaper size, and suitable type to prevent skin irritation (4), bedsores (5), and urinary tract infections (6). To use bladder diaries for diagnosis and care, periodic diaper checks are required to assess the diaper absorption conditions (7). This task is time-consuming for caregivers and stressful for care recipients (8, 9).

Accordingly, several sensor studies to assess urinary information, such as ultrasound, gas, electrical resistance, and capacitance, are being conducted globally (10–20). Toymus et al. (11) reported an integrated wearable ultrasound monitoring device for the accurate and autonomous continuous monitoring of bladder volume. The *in vivo* measurements of healthy volunteers showed a mean relative error of 11.17% for patches of various bladder shapes and volumes within 100–800 ml. Nevertheless, the device may be affected by anatomical changes around their bladder, such as bladder extensibility and ptosis in elderly people who use diapers. Therefore, it is challenging to evaluate such devices *in vivo* on numerous people, including people of various races and ages suffering from various sicknesses (12). Sugano et al. (13) reported a gas sensor that can be used with a cushion to measure excretion without touching the urine. A total of 206 data points were obtained from two groups of five subjects and three residents in a nursing home, which were divided into training and evaluation data and used to build an excretion detection model. The model achieved an accuracy rate of approximately 76%. However, the sensor cannot be used to measure urine volume. Kim et al. (14) developed a smart diaper system that measures urine volume from changes in electrical resistance by attaching two conductive materials inside diapers. They reported that the same amount of urine obtained from the weight difference of diapers measured by an electronic balance was obtained from the measurements of 97 residents in a nursing home. The system also addresses cases of multiple urinations, with the ultimate goal of calculating the volume of each urination. However, the conductive material should be disposable because measurement is possible only when the conductive material comes into contact with urine. Even if diapers were specifically developed for this sensor system, they would be more expensive than general-purpose diapers and would not be readily available. Therefore, it is desirable to develop a diaper sensor that is non-contact with urine and can measure the volume for multiple urination. Each of these urinary diaper sensor systems has its own advantages and challenges. At present, there is no universal technology, and it is hoped that a technology that is easy to disseminate will be developed.

Commercial diaper packages indicate the “approximate numbers of absorption (ANA),” such as 150 or 200 ml/urination, and the approximate number of times urine is absorbed. The Japan Hygiene Products Industry Association defines this label as an approximate absorbency factor, and it should be accompanied by the approximate number of usage, and voided volume per urination shall be stated together (21). Further, owing to competition in technological development, various diaper manufacturers have developed diapers with different product characteristics, such as absorbency and breathability (22, 23). Therefore, differences in diaper characteristics could affect the accuracy of urination measurement.

From the above, it is necessary to easily and accurately obtain diaper information as well as provide diaper care that is less burdensome for caregivers and care recipients. Ideally, the sensor should be able to signal the need for a diaper change. In response, we reported a preliminary study on a repeatable diaper sensor that can automatically inform caregivers when to change a diaper using a capacitance change and can measured urine absorption volume without contact with urine (24–26). In this study, we developed a diaper sensor that can be applied to various urination patterns of bedridden elderly people who wear diapers of different ANA and from different manufacturers as well as quantitatively evaluate the amount of urine absorbed by diapers. This paper reports the results of experiments using a phantom to verify the accuracy of our developed sensor system's evaluation of urination and a simulated experiment to evaluate the actual time and urine absorption volume.

## 2 Materials and methods

### 2.1 Overview of capacitive diaper sensor system

Capacitive diaper sensor systems used in this study were developed based on previous studies (24–26). The research design of this study was approved by the ethics committee of the University of Toyama (research number: R2018130).

The basic diaper structure comprises a surface material, a water-absorbing material, and a waterproof material (21). After passing through the surface material, urine spreads in a two-dimensional plane and is absorbed by the water-absorbing material, which is mainly a mixture of fluff pulp and a superabsorbent polymer (SAP). The systems demonstrated essential diaper characteristics, such as excreted urine retention and leakage prevention. Further, the systems combine and use outer and inner diapers together (27). Outer diapers are pants-type diapers that can be worn like underwear and tape-type diapers for those who spend most of their time in bed. For example, a pad-type diaper, which is an inner diaper, can be layered and combined with a tape-type diaper. Attaching inner diapers to outer diapers and changing them after only one or multiple incontinence cycles is a well-known practice in Japan to reduce physical and financial burdens (8).

**Figure 1:**
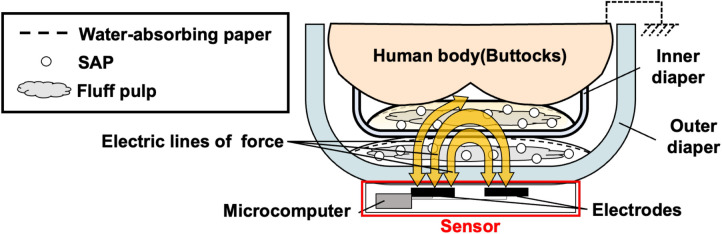
Schematic of the basic principle of the capacitive diaper sensor.

Figure 1 shows a schematic of the basic principle of a capacitive diaper sensor we have developed. Our developed capacitive sensor is worn on the outside of the outer diaper layered against the human body. It then measures changes due to urination inside the diaper by capacitance, which is an electrical characteristic. The sensor comprises electrodes and a microcomputer. The electrodes are comb-shaped to detect urination over a wide area. The entire sensor is encased in a polyethylene terephthalate film, meaning that if the sensor gets dirty, it can be reused by simply wiping the dirt off. The microcomputer (CYBLE-022001-00, Cypress Semiconductor, CA, USA) includes capacitance measurement and Bluetooth Low Energy (BLE) communication functions. Including powered by a coin battery (CR2450, Panasonic, Tokyo, Japan), the external size of the sensor circuit part is W35.0 × H12.0 × D75.0 mm. The sensor circuit part has a connector that connects a pair of electrodes. This allows the user to spend time without discomfort, even with the sensor attached. The microcomputer outputs the value of the A/D change in capacitance by the Δ*Σ* method (28), the raw count [counts]. The raw count has a negative correlation with the capacitance, i.e., the raw count decreases as the capacitance increases. To extend the battery life, 3.8 s measurements were taken approximately every 5 min. With measurements taken every 1 s, the battery life was at least 146 days. The data were stored on a Personal Computer.

**Figure 2:**
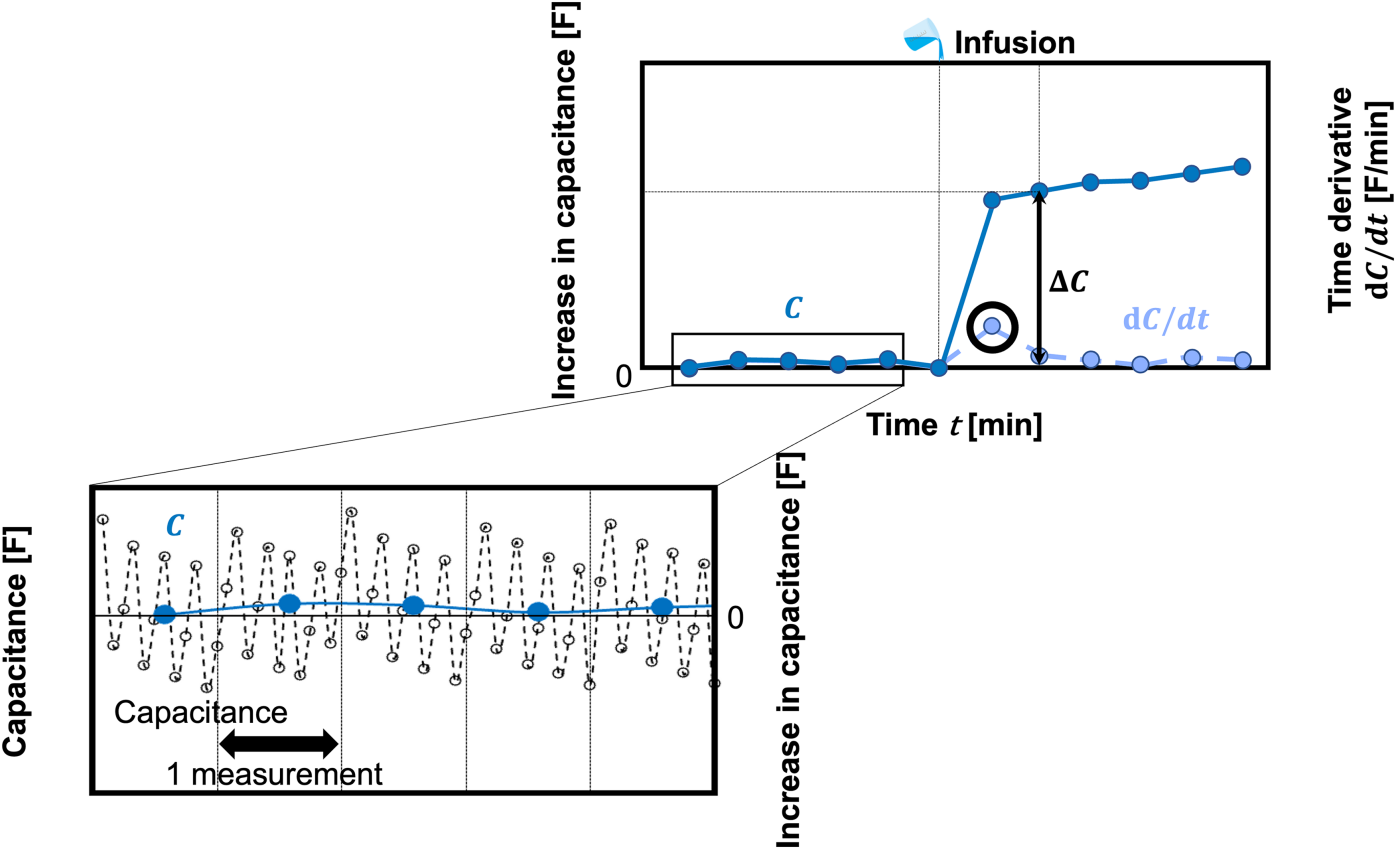
Schematic of the analysis image.

Figure 2 shows a schematic of the analysis image. Because the sensor is restarted for each measurement, a steady noise is generated. The measurement noise was stabilized by calculating the average capacitance of approximately 12 data points. These data points were measured approximately every 5 min during sensor operation. Moreover, the electrode position changes slightly with each measurement. As a result, the initial output capacitance value varies when to change the diaper using a capacitance change. Therefore, urination is evaluated by the microcomputer output, *C* [F], which is the baseline shift in capacitance from the time the diaper was worn. Immediately after urination, *C* changes rapidly. The time of urination is estimated from the time when the peak value of the time derivative waveform of *C*, d*C*/d*t*, is obtained. The amount of urine absorbed by a diaper was calculated from the transient change in *C*, Δ*C*, before and after this time. The total urine absorption volume, *V*, is obtained from the sum of the time changes, *Σ*Δ*C*, by the conversion coefficient, k:

(1) $$\begin{array}{c} V=k\;\sum \Delta C \end{array}$$

### 2.2 Phantom experiment

**Figure 3:**
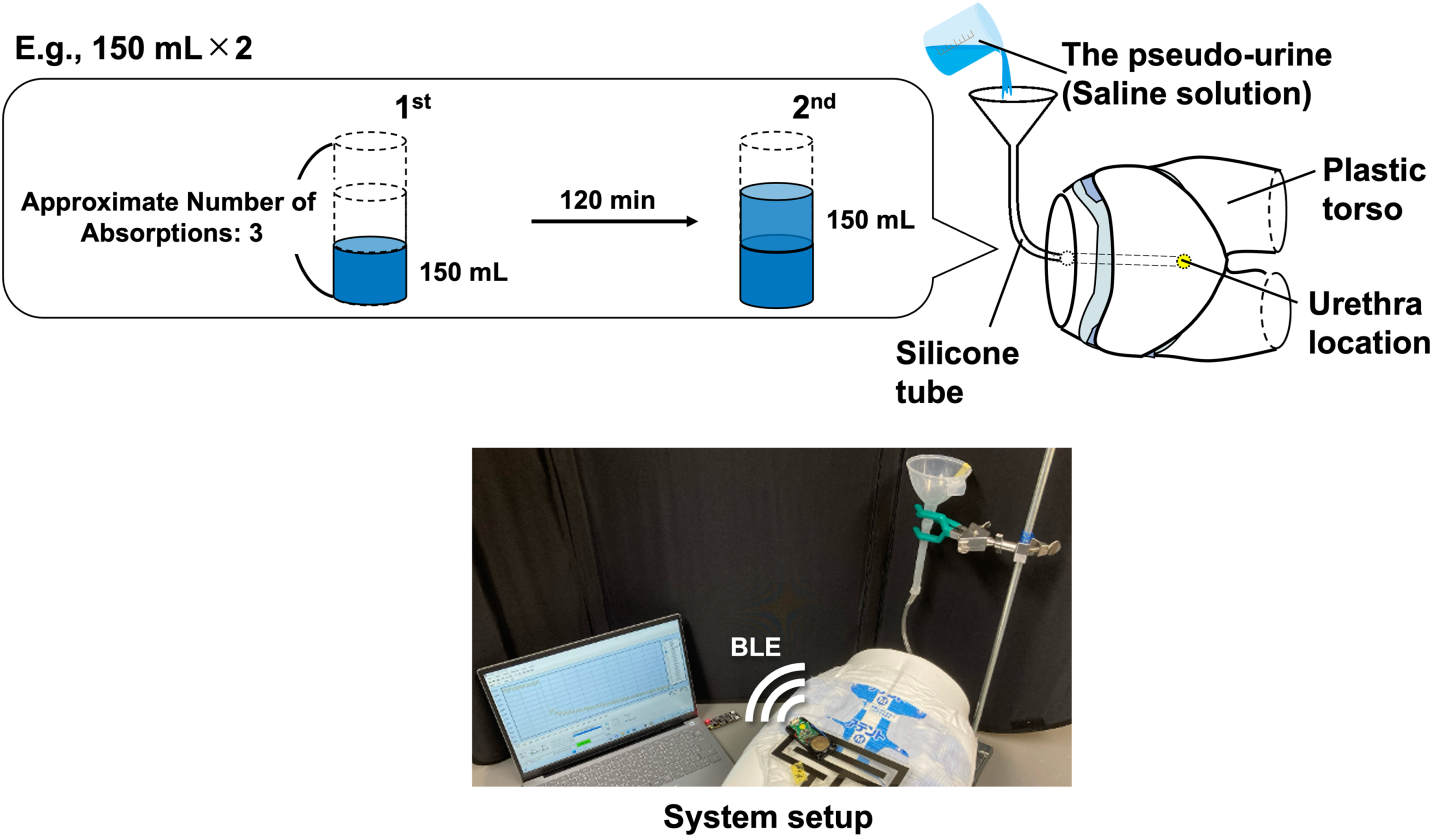
Schematic of the phantom experiment.

Three experiments were conducted using a plastic torso filled with saline as a phantom (Figure 3). The phantom was placed in the supine position, and a small hole was opened at the urethral location. Yamanishi et al. (29) reported an average flow rate of 14.0 ± 5.07 ml/s in the supine position in the measurements of 21 healthy male collaborators aged 24–40 years, with a mean age of 29 years. However, the flow rate tends to be lower in the elderly than in the young (30). Kumar et al. reported the average flow rate of 13.05 ± 6.12 and 8.9 ± 4.06 ml/s for males aged 16–50 and >50, respectively. Moreover, regardless of gender, the average flow rate of premenopausal and post-premenopausal females are 12 ± 4.6 ml/s and 10.2 ± 3.52 ml/s were reported (31). Thus, the urinary flow rates decrease with age. In this study, pseudo-urine was infused from inside the phantom at an average flow rate of 7.8 ml/s through a silicone tube with an inner diameter of 3.1 mm.

The main component of human urine is water, followed by urea, creatinine, uric acid, chloride, and sodium (32). In this study, a saline solution containing the electrolyte components chloride and sodium in the same proportion as urine was used as pseudo-urine. The saline solution was prepared by dissolving sodium chloride in deionized water to 0.9 wt%. An inner diaper, outer diaper, and capacitive sensor were attached to the phantom. The capacitive sensor encased inside the diaper cover was attached to the phantom with Velcro fasteners on the diaper cover. The experiments were conducted in a room where the temperature and relative humidity were kept constant at approximately 25°C and 60%, respectively.

#### 2.2.1 Experiment α: comparative verification by urination pattern

First, experiment α (comparative verification by urination pattern) was performed, with inner diaper: Attento, ANA: 4, Daio Paper Corp., Ehime, Japan; and outer diaper: M size, Daio Paper Corp. As mentioned in the introduction section, commercial diaper packages indicate absorption guidelines of 150 or 200 ml/urination (17); therefore, the pseudo-urine volumes of 150, 200, 250, and 300 ml were infused into the phantom only once each. Moreover, to allow for change after only one or multiple incontinence cycles, pseudo-urine was infused into the phantom multiple times. The total volume of pseudo-urine infused multiple times was 300 ml. The combinations of pseudo-urine to be infused were: (a) 100 ml × 3, (b) 150 ml × 2, and (c) 200 + 100 ml, with a 120 min interval fixed between infusions. The experiment was performed seven times for each pattern.

Furthermore, to examine the effect of pseudo-urine temperature on the evaluation of absorption, the saline solution was infused at 37°C, close to body temperature. The warm saline solution was warmed in a thermostatic water bath set to 37°C, the pseudo-urine volumes of 150 and 300 ml were infused into the phantom one times each.

#### 2.2.2 Experiment β: effect of ANA

Next, to evaluate the effect of ANA, the urination pattern experiment was performed using inner diapers from the same manufacturer but with different guidelines and ANA: 2, Daio Paper Corp. The outer diaper was the same as that used in experiment α. Pseudo-urine volumes of 100, 150, 200, and 250 ml were infused once. In addition, a multiple infusion pattern in which 100 ml × 2 was infused at a 120 min interval was considered. The experiment was performed seven times for each pattern.

#### 2.2.3 Experiment γ: evaluation by each diaper manufacturer

Finally, for comparison and evaluation by each diaper manufacturer, experiments were performed one time for each manufacturer with inner and outer diapers from five manufacturers (Table 1; A: Daio Paper Corp., ANA = 4; B: Kao Corp., Tokyo; C: Koyo Co., Ltd., Kanagawa; D: Livedo Corp., Ehime; and E: Unicharm Corp., Ehime). Measurements were compared for each manufacturer's diapers in a pattern with multiple infusions of pseudo-urine, 150 ml × 2 at a 120 min interval. For the B-diaper, experiments on additional patterns were performed to compare the total urine absorption volume in detail. Three infusion patterns were considered, including a single infusion of 300 ml and multiple infusions of 200 + 100 ml, such as the urination pattern (c) in experiment α, seven times for each pattern.

**Table 1:** List of diapers from five manufacturers.

Manufacturer (Brand name)		A		B	C	D	E
		Daio Paper Corp. (Attento)		Kao Corp. (Relief)	Koyo Co., Ltd. (Onlyone care)	Livedo Corp. (Refre)	Unicharm Corp. (Lifree)
Inner diaper, pad-type	Approximate Number of Absorption	4	2	3			
	(ANA)						
	Surface materials	Polyolefin-based nonwoven fabric				Polyolefin nonwoven fabric	Polyolefin, Polyester nonwoven fabric
	Water-absorbing materials	Fluff pulp		Fluff pulp	Fluff pulp		
		SAP		Acrylic-based SAP	SAP		
		Water-absorbing paper		Water-absorbing paper	Water-absorbing paper		
	Waterproof materials	Polyethylene films		Polyolefin-based film		Polyethylene film	Polyolefin film
	Length	56	49	48	45	48	49
	Width	30	21	20	15	21	28
Outer diaper, tape-type	Surface materials	Polyolefin-based nonwoven fabric					Polyolefin nonwoven fabric
	Water-absorbing materials	Fluff pulp		Fluff pulp	Fluff pulp		
		SAP		Acrylic-based SAP	SAP		
		Water-absorbing paper		Water-absorbing paper	Water-absorbing paper		
	Waterproof materials	Polyethylene film		Polyolefin-based film	Polyethylene film	Polyolefin-based film	Polyolefin film

### 2.3 Simulated experiment

**Figure 4:**
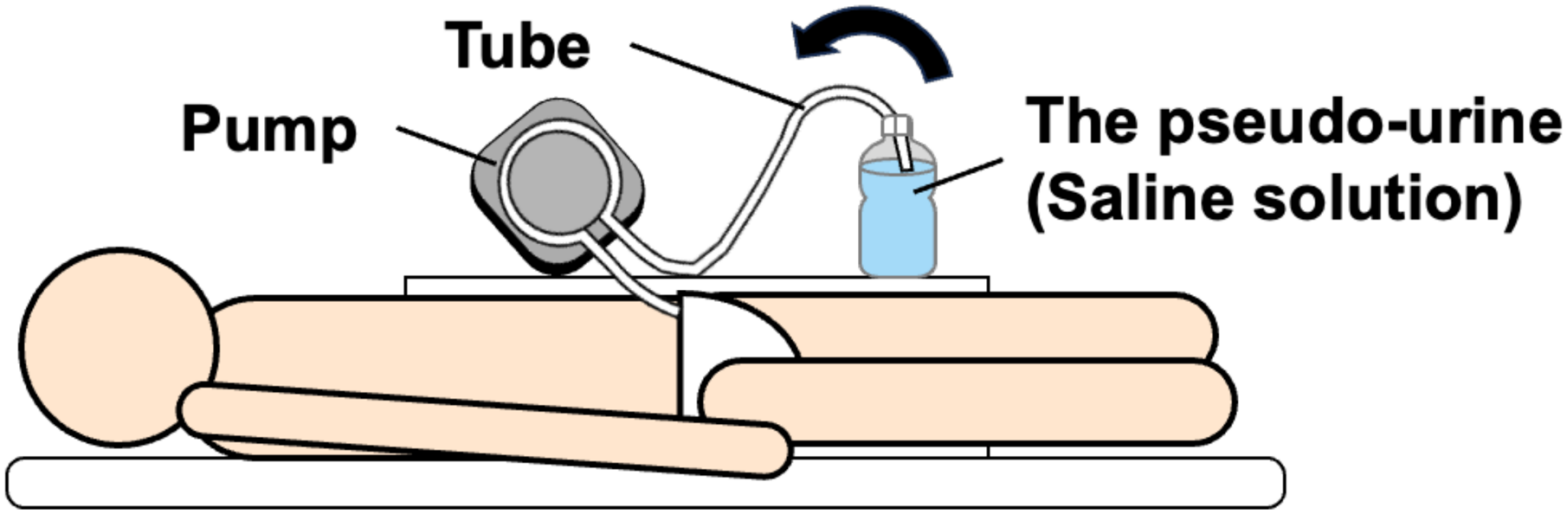
Schematic of the simulated experiment.

As shown in Figure 4, to examine the actual urination time and urine absorption volume when the human wears a diaper, one healthy male adult volunteer (24 years) wore inner (ANA = 4) and outer (A-diapers) diapers and the capacitive sensor. As in the phantom experiment α, the warm saline solution was used as the pseudo-urine. The pseudo-urine was infused through a tube (Tygon Tube, LMT-55 1/4 × 3/8 inch, ϕ6.35 × ϕ9.53 mm, Tygon). Assuming urination by the volunteer in the supine position, the pseudo-urine was infused at an average flow rate of 11.7 m/sec via a pump (Ring Pump, RP-S06N-700Z-DC24V, Aquatech Co., Ltd., Osaka, Japan). The infusion patterns were the same as in experiment γ.

## 3 Result

### 3.1 Phantom experiment

**Figure 5:**
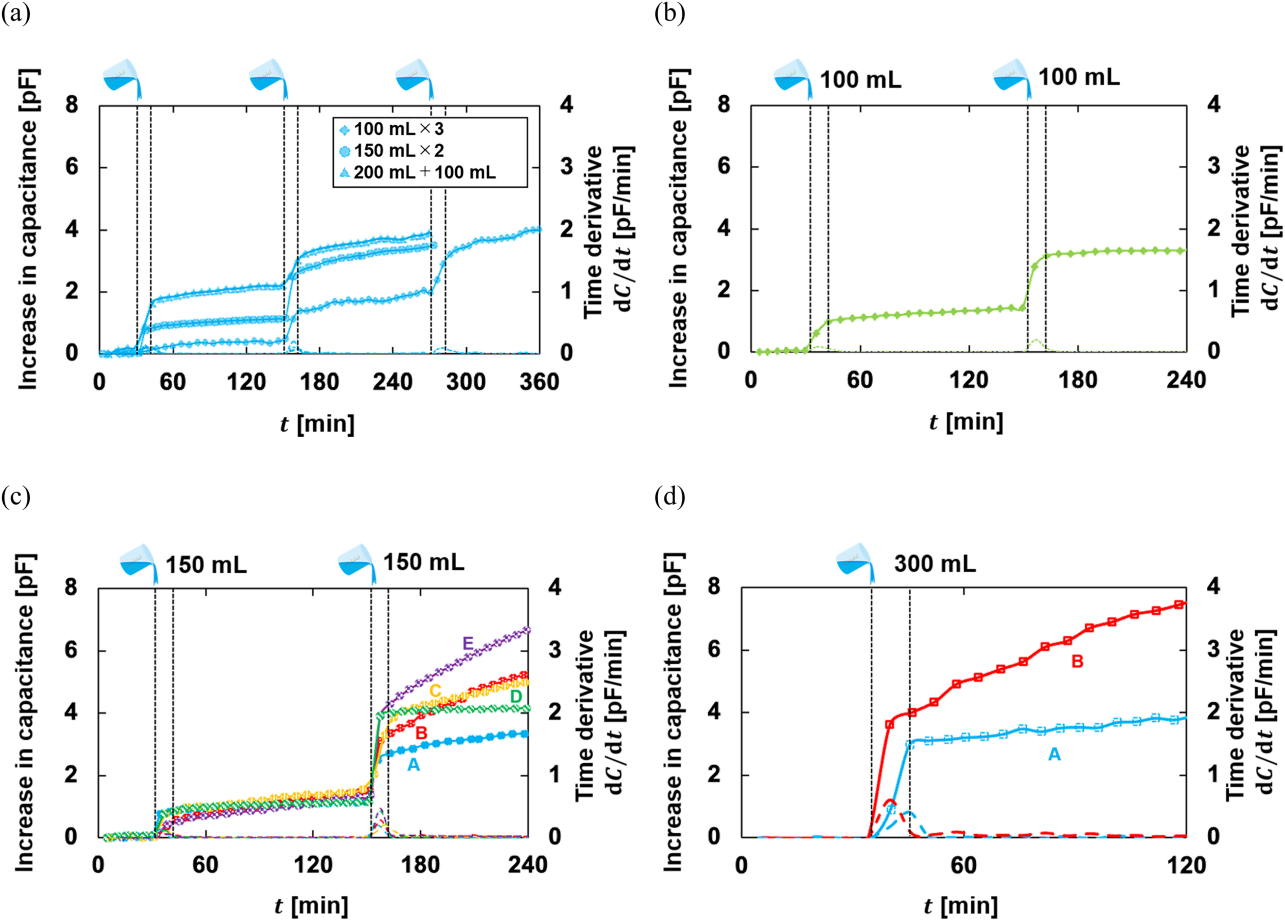
A typical phantom experiment example: **(a)** experiment α, the comparative verification by urination pattern, multiple infusions; **(b)** experiment β, effect of ANA, a multiple infusion; **(c)** experiment γ, the evaluation by each diaper manufacturer, 150 ml × 2 infused; **(d)** experiment γ, the evaluation by each diaper manufacturer, 300 ml.

**Figure 6:**
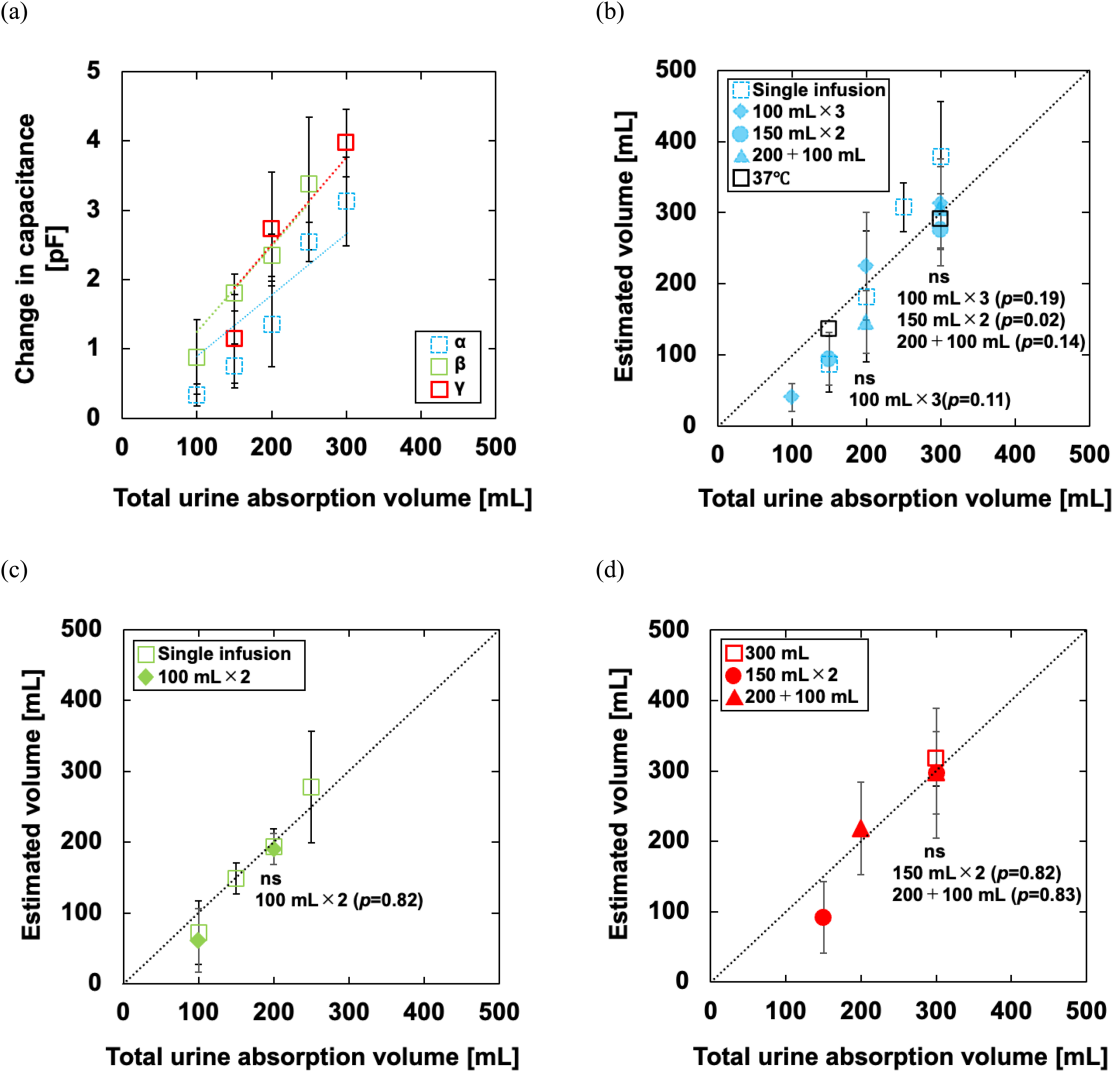
**(a)** Relationship between the total urine absorption volume and change in the microcomputer output of the phantom experiment. The dotted line is a regression line. The calculations for 150- and 200 ml infusions in experiment α and 100 ml infusion in experiment β have *n* = 14, including data from the first of multiple infusions; **(b–d)** relationship between the total urine absorption volume and the estimated volume of experiments α, β, and γ. The dotted line is an ideal straight line. The results for multiple infusions were tested for statistical significance using the Dunnett test based on the results for a single infusion. For all analyses, the significance level was set at *p* < 0.01.

Figure 5 shows a typical example of the phantom experiment. By infusing pseudo-urine, *C* increased and the infusion time can be estimated from the time indicated by the peak value of d*C*/d*t*. In the multiple infusion patterns of experiments α and β, *C* tends to the amount of increase at the second infusion was larger than the increase at the first infusion. In experiment γ, for all manufacturers' diapers, the amount of increase at the second infusion was greater than that at the first infusion. Each manufacturer exhibited different trends in the output waveforms. For B-, C-, and E-diapers, the increase over time after the second infusion was particularly large. A- and D-diapers did not show much change over time after infusion. However, the time when pseudo-urine was infused was identified from the time derivative of the output regardless of the manufacturer. In addition, the increase over time became larger as the volume increased when comparing A- and B-diapers. Figure 6 shows the mean and standard deviation of the analysis results. The slopes of experiments α, β, and γ were 0.0089, 0.0124, and 0.0125 pF/ml, respectively. Taking the inverse of these values, the conversion coefficients were calculated as 112.4, 80.6, and 80.0 ml/pF, respectively.

Experiment α, a comparison of infusion patterns, showed a good relationship in total change regardless of the pattern. This relationship was observed for the warmed saline solution as well. The multiple-infusion results are consistent with the single-infusion results. When the conversion coefficient was used to estimate the urine absorption volume of single infusions of 150, 200, 250, and 300 ml, the mean absolute percentage errors (MAPE) were 42.7 ± 24.7, 43.3 ± 18.0, 23.0 ± 13.9, and 30.1% ± 21.2%, respectively. Further, as the volume of multiple infusions increased, the MAPE tended to decrease to 59.5% ± 19.6%, 33.1% ± 21.8%, and 17.7% ± 11.7% in (a) 100 ml × 3, respectively. The MAPE was 36.9% ± 24.9% and 12.2% ± 13.9% in (b) 150 ml × 2 and 26.9% ± 22.1% and 18.4% ± 6.6% in (c) 200 + 100 ml. The comparison results in experiment β showed differences in the output value. Nevertheless, the urine absorption volume of single infusions of 100, 150, 200, and 250 ml was evaluated with MAPE of 47.9% ± 29.7%, 13.2% ± 5.8%, 10.4% ± 7.7%, and 23.4% ± 23.8%, respectively. For multiple infusions, the MAPE of the first infusion was estimated to be 44.6% ± 17.2% and that of the second infusion was 9.3% ± 5.5%.

In experiment γ, the MAPE for the single-infusion pattern of 300 ml was 11.5% ± 8.3%. In the multiple-infusion pattern of 150 ml × 2, the MAPE was 43.8% ± 27.5% and 12.2% ± 13.9% for the first and second infusions, respectively. In the multiple-infusion pattern of 200 + 100 ml, the MAPE was 59.2% ± 22.2% and 16.9% ± 9.8% for the first and second infusions, respectively.

### 3.2 Simulated experiment

**Figure 7:**
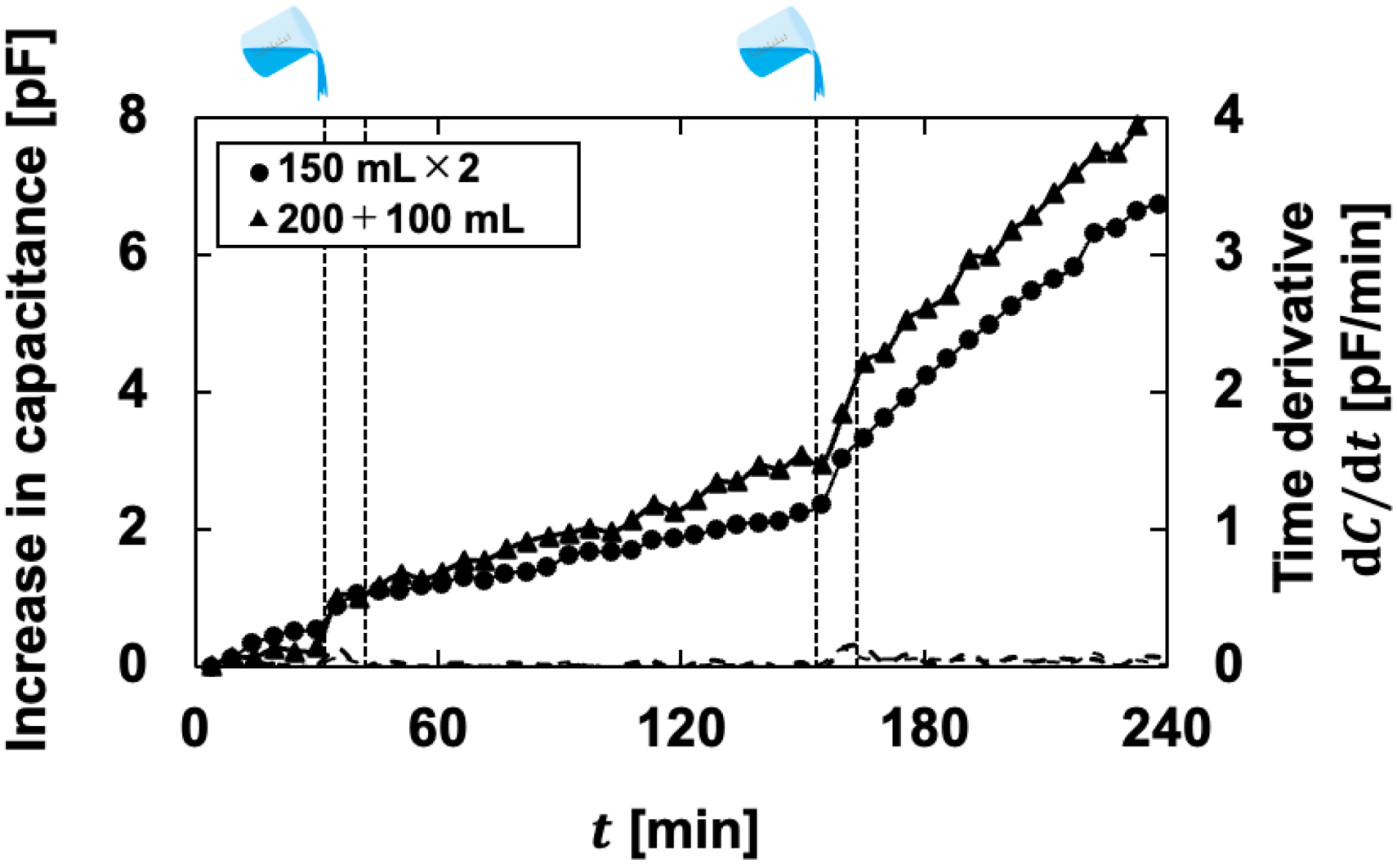
Comparison of a typical example of the phantom and simulated experiments.

**Figure 8:**
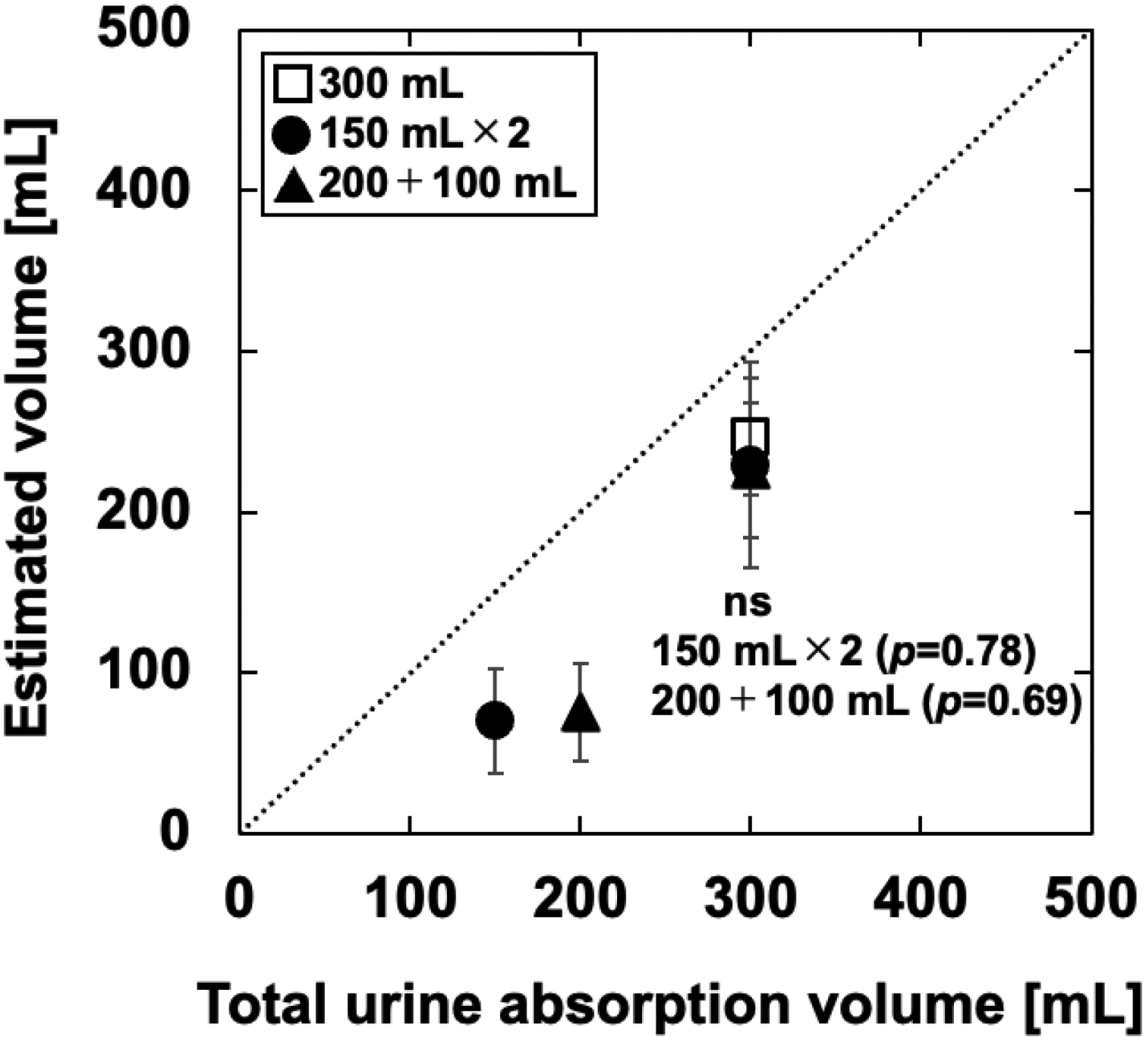
Relationship between the total urine absorption volume and the estimated volume of the simulated experiment. The dotted line is an ideal straight line. The results for multiple infusions were tested for statistical significance using the Dunnett test based on the results for a single infusion. The significance level was set at *p* < 0.01.

As shown in Figure 7, the output waveform changed over time as in B-, C-, and E-diapers, despite the use of A-diapers. The estimated volume was underestimated regardless of the urination pattern, although the results were generally consistent with the estimated volume using the conversion coefficients obtained from the phantom experiments (Figure 6). Figure 8 shows the relationship between the total urine absorption volume and the estimated volume of the simulated experiment. The MAPE for the single-infusion pattern of 300 ml was 19.4% ± 9.2%. In the multiple-infusion pattern of 150 ml × 2, the MAPE was 53.3% ± 21.8% and 26.9% ± 16.9% for the first and second infusions, respectively. In the multiple-infusion pattern of 200 + 100 ml, the MAPE was 62.4% ± 15.4% and 24.7% ± 14.1% for the first and second infusions, respectively.

## 4 Discussion

The evaluations performed in this study by experiments α, β, and γ exhibited differences in the conversion coefficients, especially for experiment α. Therefore, it is necessary to adjust the sensitivity for each diaper type.

In the statistical analysis, the multiple-infusion pattern of 150 ml × 2, based on 300 ml in experiment α, *p* = 0.02. The biggest problem with diaper care, for both the person and the caregiver, is urine or stool leaking out of the diaper and staining clothing and bedding. To prevent this, early diaper changes are effective. Although *p* = 0.02, the estimated volume is underestimated. This helps prevent the biggest problem with diaper care. To avoid saturating the localized areas of water-absorbing materials, diapers have excellent diffusion properties, so that urine is absorbed evenly in other areas (33). The sensor captures the area where the wetted portion overlaps with electrodes covering the diaper. Therefore, the amount of change tends to be larger at the second time of urination than at the first urination because the increase rate in the wet area is larger at the second urination. The influence of sensor resolution on measurement accuracy has been minimized through noise reduction. Regarding variations in diaper structure, since the diapers used are industrially manufactured products, their structural differences are within an acceptable range and are not expected to significantly affect the measurements. Inconsistencies in infusion speed could introduce some error; however, our analysis suggests that these variations have less impact compared to how urine spreads within the diaper. The urine spreads through the absorbent material appears to be the most influential factor affecting the measurement accuracy.

In experiment γ, the difference in the output waveforms over time among the manufacturers was observed. The five manufacturers' diapers used in this study used various surface materials, such as polyolefin-based nonwoven fabric and polyester nonwoven fabric. The water-absorbing materials did not differ significantly. As for the waterproof material, it is thin enough so that its effect by capacitance changes can be very difficult. Each diaper manufacturer designed various diaper materials, including deodorant performance and moisture release for comfort. Therefore, this slight difference in material may have resulted in a change in capacitance over time after urine absorption.

In the simulated experiment, the volume tended to be underestimated compared with experiment α. The warmed pseudo-urine cools down after some time has passed since the pseudo-urine was infused (15). This effect of urine temperature was considered negligible in the analysis method of this system. In the experiment, the phantom temperature was about room temperature. Therefore, in the simulated experiment, the output waveforms were considered to have changed over time due to heat dissipation from the body temperature. Further, a possible reason for the absorption volume underestimation is that the dielectric constant of the human body, which comprises living tissue such as skin, muscle, and blood, is higher than that of a phantom internally filled with saline. Because this sensor system analyzes the sum of changes during urination, it can make quantitative evaluations that approximate urine absorption volume without being affected by the human body. To the best of our knowledge, there are few reports in Japan on actual urinary information of the bedridden elderly (34, 35). Electronic recording of urination, as in this study, is expected to be used to predict caregiving activities to a certain extent (9, 36).

In previous studies (24–26), we have already reported a preliminary experiment in which we assumed that a small volume of urine was excreted in a short period of time. As a result, urine tends to be similar to a single urination because of the short intervals. This measurement was performed using a single diaper combination. Therefore, the accuracy of measurement by urination pattern, ANA, and the manufacturer was not investigated. In this study, diapers from five different manufacturers were investigated. The total capacitance change due to urination is used to inform when it is time to change the diaper. For hygienic reasons, diaper changes should be performed when the diaper still has the absorptive capacity to prevent the biggest problem with diaper care. It was indicated that total urine absorption volume can be informed when it is time to change diapers using a sum of capacitance changes during urination. As long as the spreading of urine is irregular, it is inevitable that errors will increase, especially when urinating into a dry diaper. However, we consider this acceptable in informing people when to change their diapers. For example, 150 ml/urination is used as a guideline and ANA: 4 is used, half of the estimated maximum absorption of 600 ml is 300 ml. The sensor was able to quantitatively detect that half of the ANA had been exceeded. As a result, there is a high probability that this sensor could also contribute to the notification of the appropriate time to change a diaper, which is the most important factor in diaper care.

Nevertheless, this study has several limitations. First, the possibility of false detection due to body movement: because measurements were performed only in the supine position, it is necessary to verify whether evaluation for changes in body position is possible. Second, the use of larger diapers than those used. The sensor requires only a pair of simple electrode shapes and a minimal circuit in this study. On the other hand, it is difficult to adapt them to fit a wide size of diapers when urine diffusion is taken into account. The sensor needs to be optimized to fit larger diapers, such as using matrix electrodes (37). Using matrix electrodes, the direction of urine absorption could be estimated. Third, the method for securing the sensor: the diaper cover used in this study does not take full advantage of the highly breathable diaper's performance. We will explore alternative attachment methods that maintain both comfort and sensor functionality without compromising the ease of use for those requiring care, for example, by using conductive fibers with thin fabrics (16, 17). Lastly, the method of functions obtained from sensors, including RFID, NFC, and other communication technologies and energy supply, must be considered (18–20).

## 5 Conclusion

We developed a sensor that is compatible with diapers from multiple manufacturers, detects multiple urination events, and measures accumulated urine absorption. Using capacitance changes to decide when to change the diaper. Three phantom experiments and one simulated experiment were performed to evaluate the system's accuracy in detecting urine for various urination patterns. The system can be used to approximate and quantify the urine absorption evaluation without being affected by the human body. In addition, the system's ability to accommodate several diaper products makes it highly practical and easy to integrate into existing care routines.

## Data Availability

The datasets presented in this article are not readily available in order to protect a patent. Requests to access the datasets should be directed to d2372001@ems.u-toyama.ac.jp.
